# Mitochondrial Replacement Therapy: Halachic Considerations for Enrolling in an Experimental Clinical Trial

**DOI:** 10.5041/RMMJ.10216

**Published:** 2015-07-30

**Authors:** Rabbi Moshe D. Tendler, John D. Loike

**Affiliations:** 1 Rosh Yeshiva, Rabbi Isaac Elchanan Rabbinical Theological Seminary, the Rabbi Isaac and Bella Tendler Professor of Jewish Medical Ethics and Professor of Biology, Department of Biology, Yeshiva University, New York, USA; 2 Director of Special Programs, Center for Bioethics, Columbia University, New York, USA

**Keywords:** Bioethics, clinical trials, halacha, mitochondrial replacement therapy, social responsibility

## Abstract

The transition of new biotechnologies into clinical trials is a critical step in approving a new drug or therapy in health care. Ethically recruiting appropriate volunteers for these clinical trials can be a challenging task for both the pharmaceutical companies and the US Food and Drug Administration. In this paper we analyze the Jewish halachic perspectives of volunteering for clinical trials by focusing on an innovative technology in reproductive medicine, mitochondrial replacement therapy. The halachic perspective encourages individuals to volunteer for such clinical trials under the ethical principles of beneficence and social responsibility, when animal studies have shown that health risks are minimal.

## INTRODUCTION

Reproductive medicine and genetic engineering are emerging biotechnologies that hold the promise of improving human health in unimaginable ways by potentially allowing scientists to correct any of the over 6,000 genetic mutations that are responsible for human diseases. Here we highlight one recent innovative technology that combines reproductive medicine with genetic engineering, called mitochondrial replacement therapy (MRT), and discuss its halachic implications. Specifically, we address two major halachic issues: if and when it is appropriate to volunteer for phase I clinical MRT trials.

## THE IMPORTANCE OF MITOCHONDRIAL REPLACEMENT THERAPY

One important objective from decades of genetic research is developing safe and effective methods to transfer genetic material from one cell into an egg to create a viable and healthy embryo. Mitochondrial replacement therapy could potentially be a safe and effective technology that allows women who have mitochondrial mutations to have healthy children.

In the USA about 12,000 women have a genetic mutation in their mitochondrial DNA. The clinical manifestations in severe cases of mitochondrial diseases include heart disease, liver disease, muscular dystrophy, respiratory problems, and, in rare instances, death. Without intervention, mitochondrial DNA mutations will be passed on to all children of the affected woman. However, clinical trials are about to begin in various countries, including the United States (US) and Great Britain, to use MRT to provide these women with the opportunity to have healthy children.

Several protocols are being considered for MRT that all involve the use of genetic materials from three genetic donors to create an embryo in the laboratory.^1^ Maternal spindle transfer, on which this paper will focus, was first described as a method for MRT in 2009.^2^ (Another method being considered is called pronuclear transfer; for a description of that method see Craven et al. 3) The first step in the maternal spindle transfer MRT method is to obtain an egg from a donor woman who does not have mitochondrial disease. The nuclear DNA is removed and discarded from that donor egg without removing any of its healthy mitochondria. Then, an egg is retrieved from a woman with the affected mitochondrial disease, and the nucleus containing the chromosomal genetic material is retrieved from the egg and transferred into the donor egg. The reconstructed egg is then fertilized using sperm from the husband of the affected woman and is implanted into the uterus of the affected woman to gestate into a “healthy child.”

Without MRT, a woman with a family history of mitochondrial diseases or a female who spontaneously develops a mitochondrial mutation will have only two options in order to have healthy children. One option is to use a donor egg from a healthy mitochondrial DNA donor and then fertilize it with the sperm from the husband of the affected woman; the affected woman can, if all parties agree, gestate that embryo to term. The second option is for the affected woman to adopt a healthy genetically unrelated child.

In principle, MRT holds great promise for success. Ten years ago a form of MRT was used to allow women with mitochondrial mutations to have healthy children. However, for unknown reasons that may not be directly related to this technology a few of the children were born with serious birth defects, such as Turner’s syndrome. New animal studies using MRT and more experience with *in vitro* fertilization (IVF) technology have led scientists in Great Britain to become more confident in this therapy and to begin recruiting volunteers for a clinical trial to assess the efficacy and safety of MRT.

### FDA-Sponsored Clinical Trials

Before discussing the halachic ramifications of this technology it is important to review how the Food and Drug Administration (FDA) in the US has structured various phases of clinical research before a new protocol or drug is approved in the US. Phase I studies are usually conducted using healthy volunteers. The overall objective of phase I studies is to identify the most frequent side effects of a drug or protocol. The number of subjects enrolled in phase I trials typically ranges from 20 to 80. Phase II studies focus on the clinical effectiveness of a drug or protocol and are started after phase I studies reveal relative safety with no unacceptable toxicity. Phase II studies are designed to obtain preliminary data on whether a drug or protocol exhibits any efficacy in people who have a specific disease. Safety continues to be evaluated in this phase of study, and short-term side effects are again studied. Typically, the number of subjects in phase II studies ranges from a few dozen to about 300. Phase III studies are only initiated when the evidence of clinical effectiveness or benefit is demonstrated from the phase II studies. Phase III studies are designed to gather more information about safety and effectiveness, studying different populations and different dosages and using the drug or protocol in combination with other drugs or protocols. The number of subjects enrolled in phase III trials typically ranges from several hundred to several thousand.

Regarding phase I studies, it is also important to differentiate between three types of volunteers who enroll: (1) healthy volunteers with no known medical conditions related to the clinical trial; (2) volunteers who have medical conditions related to the focus of the clinical trial but where no effective treatments are available; and (3) volunteers who have medical conditions for which effective alternative treatments are available.

In MRT, two groups of volunteers need to be enrolled: women without mitochondrial disease who are willing to donate their eggs, and women with mitochondrial mutations whose eggs will receive healthy mitochondria from the donors. In addition to monitoring the health of these women, the focus of these studies will also assess the health of the children produced via IVF and MRT. Unlike the subjects in many other clinical studies, these embryos cannot provide informed consent for these studies.

All FDA-sponsored clinical trials require a control group where the volunteers do not receive the intervention. While theoretically those enrolled in the control group should not experience any benefit, subjects who are part of the control (untreated group) have claimed to have medical benefits beyond expectations.^4^ The main reasons for this salubrious experience is the placebo effect. That is, patients who think that they will improve actually demonstrate an improvement.^5^ In addition, volunteers who understand the placebo effect may still enroll in clinical trials because they often receive improved medical care (frequent visits to specialists, state-of-the-art diagnostic and imaging tests, vigilant follow-up) that can improve their overall health. It will be difficult to design control groups for MRT clinical studies.

### Health Risks in MRT Clinical Trials Participation

A major concern in volunteering for any type of clinical trial is the health risks that may develop from administering the drug or protocol being tested. Some scientists are especially cautious to begin MRT trials because science has not extensively studied whether there are health risks resulting from potentially negative interactions between the donor mitochondria DNA and recipient nuclear DNA. Secondly, in more than 50% of animal studies using MRT, faulty mitochondrial DNA was also trans ferred during the procedure, and its health effects have not been adequately studied. However, a recent study applying MRT in monkeys showed no birth defects associated with this protocol. Third, scientists and ethicists are also concerned that MRT may alter the personality of the child. There are a few published studies that indicate that genetic variations in the mitochondrial DNA influence an individual’s personality. Gardner et al., for example, reported that mitochondrial dysfunction is associated with vulnerability to psychopathology in selected patients.^6^ Mamdami et al. reported that mitochondrial dysfunction is a clinical signature in subjects with schizophrenia.^7^ Currently, many scientists believe that MRT crosses an ethical boundary because these genetic alterations will be passed on for generations.^8^

### Incentives for Entering Clinical Trials

Each year thousands of both healthy and ill people enroll for phase I clinical trials. There are many reasons why healthy people enroll in phase I trials, with financial incentives being the most cited motivation.^9^ Financial rewards in enrolling in phase I clinical trials can be significant.^10^ Often these volunteers receive financial payments for participation ranging from a few hundred to a few thousand dollars. Professional research participants have developed their own social networks, web pages, associations, and publications, which they use to learn about new studies, share information and experiences, and understand the ethical, regulatory, and scientific aspects of clinical research.^11^ Ninety percent of repeat volunteers listed financial reward as a primary motivation to enroll in clinical trials.^12^

Other reported motivations include the altruistic benefit to society, accessing ancillary health care benefits, scientific interest or interest in the goals of the study, and curiosity.^13^ People with known medical conditions consider participating as a means of monitoring a disease, the ability to take control of their lives through actively participating in the trial,^14^ and the hope that the trial will improve their medical condition. This is especially true in cancer patients who enroll in clinical trials.^15^ Many of the motivations cited above also apply to women who would consider donating their mitochondria for a clinical study on MRT. In reference to women who have mitochondrial mutations, the main reason to volunteer is to generate a healthy child.

## HALACHIC ANALYSIS OF VOLUNTEERING FOR A CLINICAL TRIAL

### Potential Health Risks

As new biotechnologies enter clinical trials, one needs to address the halachic issues related to someone who wants to volunteer for any phase I clinical trial and in particular MRT. One of the main concerns in volunteering in a clinical trial relates to the halacha that it is forbidden for a Jew to place his or her life or health in an unreasonably dangerous situation.

In addition, the Rambam states that a person must live a lifestyle that preserves health, and the Talmud and the Codes of Halacha mention a wide variety of other examples of activities that promote well-being and curtail health risks. These include:

1. eating healthily and not becoming overweight,
2. not putting one’s mouth directly onto a pipe in order to drink water,
3. not drinking water drawn from a river at night when one is unable to inspect for parasites or drink liquids that have been left exposed and unattended where there is a possibility, albeit remote, that a snake may have deposited venom,
4. not eating food that might be tainted or poisoned etc.,
5. not engaging in any activity that causes physical wounds or injuries to oneself.^16^–^18^

Endangering one’s own life is not uniformly prohibited. There is a Biblical duty to rescue someone from danger, such as rescuing someone from drowning or from a wild animal or from robbers.^19^,^20^ Moreover, we are Biblically commanded, “Do not stand by idly over your friend’s blood” (Leviticus 19:16). An underlying question in this halacha is “what degree of danger can a person incur to save another life?” The Netziv in He’emek Sh’ailah #129 states that a person cannot place him/herself in serious danger even to save another person. However, one is permitted to accept a modicum of danger as an act of beneficence (*middas chassidus*). However, there must be careful consultations with physicians and halachic ethicists to determine the modicum of danger that is allowed for each specific case. (For more information considering risks and dangers related to health care situations, see Immanuel Jakobovits.^21^)

The major health risk to women volunteering for MRT is related to ovarian hormonal stimulation to retrieve their eggs. Since most women do not present with serious side effects resulting from ovarian hormonal stimulation, volunteering for a clinical trial as a mitochondrial donor even though they may not directly benefit would certainly be considered an act of beneficence, a great *chesed*, and is permitted. Similarly, it is a great *chesed* for women with mitochondrial mutations to volunteer in MRT trials in order to have healthy children. In volunteering for such trials, these women enter into the realm of a potentially real reward—the birth of a genetically related healthy child.

What about risking health for the financial gains gleaned when healthy individuals enroll in a clinical trial? This issue also is relevant when choosing a career. Is there a difference when a person chooses a career with health risks (such as being a fireman or policeman) and volunteering to enroll in a clinical trial? We maintain that, in both situations, financial gain provides a valid halachic reason either to choose a dangerous career or to volunteer to enroll in a clinical trial. One is also halachically permitted to enter into a clinical trial for financial reasons. The risks to one’s health for financial gains are no different than choosing a career that involves an increment of risk and danger. Financial gains from a chosen employment such as building bridges, constructing underground tunnels, or physicians choosing to specialize in infectious diseases are all halachically permitted, despite the associated dangers of these professions.^22^,^23^

Since halacha provides a healthy person with the autonomy to volunteer for a clinical trial, individuals who present with medical conditions where no alternative therapies exist may surely volunteer to engage in new clinical trials, even when there may be a great medical risk. Thus, individuals with valid health or financial considerations would be permitted to volunteer in a clinical trial even when there may be a risk of potential harm. Since the health risks of MRT are not fully understood, halacha provides infertile women the autonomy to engage in these technologies in order to have healthy children. However, these women must fully understand the risks and benefits of this technology.

### Halachic Reasons to Participate in Clinical Trials

There are both positive and negative halachic reasons to volunteer for clinical trials. The Midrash states “Before the Torah was given, the Creator required that people contribute to the formation of a viable society.”^24^ This is a pre-Biblical commandment. God did not create a world to be empty but to be populated.^25^ However, in order to fulfill the will of God to populate a functioning world, society must establish water supplies and sewer systems as part of the mitzvah to create a functioning society. This theme is reflected in the Midrash’s understanding of Tehilim (Psalms 50:21) “He who offers a *korban todah* [a thanksgiving offering] brings honor to God.” The Midrash provides four examples of what is considered an offering of thanks: (1) people who remove obstacles from roads to allow fluid travel, (2) people who light candles in public so that others can travel safely at night, (3) merchants who make the effort to tithe their produce before selling to the public, and (4) people who agree to become teachers of young children—a career that is quite challenging and difficult. None of these examples mentioned in the Midrash are classical ritual actions but are activities that reflect social responsibilities.

Moreover, the Torah teaches the virtues of contributing to the general society in relating two stories of our forefathers, Avraham and Yaakov. When Avraham returned to the Land of Canaan from Egypt the Talmud states that he would stay overnight in the same lodgings that he stayed in when he first went down to Egypt.^26^,^27^ The lesson here is to “teach the proper way of conduct” that changing lodgings might be viewed as dissatisfaction with the hotel in which Avraham stayed when he first went to Egypt. In order for hotels to be successful they require a loyal clientele. It is therefore our social responsibility that we support good hotels to ensure a viable system of travel. The second example is related to Yaakov when he came to Shechem and the verse states, “he encamped before the city.”^28^, The Talmud states that Yaakov engaged in social responsibility to the members of Shechem by teaching them how to: (1) organize a currency system, (2) establish a market place to sell goods, and (3) build bath houses.^29^ Yaakov did not teach the people of Shechem ritual observances but rather social systems to benefit the municipality. These lessons that we learned from our forefathers highlight how engaging in social responsibility, such as volunteering in MRT studies, brings honor to God. In addition, there is a real need for Jews to develop friendly relations with members of other faiths in a world where so many ethnic groups live together.

The negative halachic aspect of volunteering for clinical trial may fall under the prohibition of *aiva (*anti-Jewish enmity). Failure to participate in social responsibility can incur the disdain of society. This disdain can also incur anger by members of the society that could lead to jeopardizing the health care or even lives of Jews. The halachic ruling that a Jewish physician should transgress the shabbath in treating members of other faiths is based upon the concern that failure to treat non-Jews by Jewish physicians could have severe repercussions and might even endanger Jews in the community.^30^

One example of the consequences of *aiva* is organ transplantation. Since a significant percentage of orthodox Jews believe that death is defined by cessation of heart beats, they cannot serve medically as heart donors. In many countries, anyone who is not an organ donor has a lower priority in receiving organ transplants. Living American organ donors, for example, who later need kidney transplants have much shorter waiting times, and they receive higher-quality kidneys compared with similar people on the waiting list who did not designate themselves as organ donors.^31^ In countries such as Israel and Singapore, registered organ donors are given priority on organ waiting lists. This provides an incentive for organ donor registration and has the potential to increase the pool of deceased donor organs.^32^ Moreover, as a consequence of halachic scholars not accepting brain stem death, the refusal to donate organs makes it difficult for Israelis to receive critical organs.^33^

As stated above, volunteering for clinical trials represents an act of beneficence (*chesed*) and encompasses a moral obligation of a Jew to contribute to improving the health care of a society and thus bring honor to our God. Thus, encouraging Jews to volunteer for MRT trials is quite important in a society where Jews and non-Jews interact in almost every aspect of life. In addition, volunteering for such trials would prevent enmity and promote good will. While the number of Jews who would volunteer for any phase I clinical trials is far fewer than those considering organ donation, the current atmosphere in the United States and other Western countries views orthodox Jews under a microscope for impropriety. *Aiva* is more than merely the avoidance of actions that may lead to enmity but encompasses preventative actions as well. The act of orthodox Jews participating in a socially appropriate activity adds *kavod* and pride to our people and mitigates *aiva*. In the case of MRT, there are no real medical alternatives that can produce a genetically related healthy child from a woman with mitochondrial mutations. Volunteering to be an egg donor in this case, even for financial reasons, would be encouraged and halachically permitted.

The verse that is quoted in *birchat hamazon* (“ 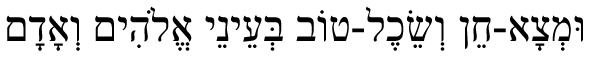
” [“So shalt thou find grace and good favor in the sight of God and man”]) alludes to the theme that Jews should engage in activities that promote good will and social responsibility.^34^ The verse suggests that the actions an individual performs should be those that find grace and sound judgment in the eyes of God and humankind. Here the term *adam* represents universal human beings, not just members of B’nei Israel. Thus, in this *bracha,* we ask God to help us act in ways that achieve the approval of God and society.

### Halachic Issues Related to the Newborn Child

There are, of course, halachic considerations concerning the newborn that emerge from such mitochondrial replacement therapies. First, how does halacha view the fact that the child was created using three genetic donors? This issue has been discussed in greater detail in a previous publication regarding human cloning, claiming that all three genetic donors may have some halachic status.^35^,^36^ One might, however, argue that MRT differs from traditional cloning in that the mitochondrial donor is only supplying an extremely small amount of DNA (37 genes out the over 20,000 genes in the human genome) to the embryo. The response to this argument might hinge on the fact that the mitochondrial DNA is critical to life. An embryo cannot develop into a child without mitochondrial DNA, and this simply cannot be ignored despite the fact that mitochondrial DNA only contributes a fraction of the genetic information.

There are other complex issues related to MRT that must be evaluated in the future. What is the religious status of child, for example, when one of the female donors is not Jewish? In halacha, religion is maternally transmitted. If an embryo is conceived using DNA from two women, do both have to be Jewish for the child to be considered Jewish? Another example relates to *pidyon haben.* Normally, the first male child born from a mother who is a *bat-Levi* or *bat-Kohen* does not require a *pidyon haben*. What about a child born from a mother who is a *bat-Kohen* or *bat-Levi* but received its mitochondrial DNA from a donor who is a *bat-Yisrael*? Similarly, would a child born from a *bat-Yisrael* require a *pidyon haben* when the mitochondrial donor was a *bat-Levi* or *bat-Kohen*? A third halachic question is whether a MRT-generated child can marry a direct relative of the mitochondrial donor? Halacha has specific criteria permitting or prohibiting marriage to a relative. All these halachic issues are quite complex and will require halachic analyses beyond the scope of this article. We hope to address them in future articles (YY’H).

## CONCLUSIONS

New biotechnologies such as gene repair^37^ and MRT are extremely complex biological processes that require a sophisticated scientific background to understand the intricate details of these technologies. Regrettably few of our halachic decisors have the scientific background to render a *p’sak* on these issues. Therefore, it is imperative that our halachic decisors consult with orthodox Torah-educated scientists who are experts in these technologies before addressing the halachic issues that are associated with gene repair and MRT. We appeal to those scientists to contribute their knowledge through lengthy and time-consuming efforts and devote the time to interact with the halachic authorities.

As clinical trials using MRT are about to begin, we present in this article one important halachic consideration whether one is permitted to volunteer for such MRT trials or any clinical trial. In general, halacha permits women to engage in new biotechnologies such as MRT to have healthy offspring. Here, we advocate that volunteering for such trials is not only a *chesed* but engenders social responsibility so that Jews are contributing to the overall health of our society. Only through clinical trials can we establish whether MRT will afford women with mitochondrial mutations the opportunity to have healthy children.

## Abbreviations

FDA: Food and Drug Administration
IVF: *in vitro* fertilization
MRT: mitochondrial replacement therapy
US: United States.
